# Real-world goal-directed behavior reveals aberrant functional brain connectivity in children with ADHD

**DOI:** 10.1371/journal.pone.0319746

**Published:** 2025-03-18

**Authors:** Liya Merzon, Sofia Tauriainen, Ana Triana, Tarmo Nurmi, Hanna Huhdanpää, Minna Mannerkoski, Eeva T. Aronen, Mikhail Kantonistov, Linda Henriksson, Emiliano Macaluso, Juha Salmi

**Affiliations:** 1 Department of Neuroscience and Biomedical Engineering, Aalto University, Espoo, Finland; 2 Department of Computer Science, Aalto University, Espoo, Finland; 3 Child Psychiatry, University of Helsinki and Helsinki University Hospital, Helsinki, Finland; 4 New Children’s Hospital, Pediatric Research Center, Helsinki, Finland; 5 Faculty of Biological and Environmental Sciences, University of Helsinki, Helsinki, Finland; 6 Lyon Neuroscience Research Center, Lyon, France; 7 Aalto Behavioral Laboratory (ABL), Aalto University, Espoo, Finland; 8 AMI-centre, Aalto University, Espoo, Finland; 9 MAGICS, Aalto Studios, Aalto University, Espoo, Finland; 10 The Research Center for Psychology, Faculty of Education and Psychology, University of Oulu, Oulu, Finland; Museo Storico della Fisica e Centro Studi e Ricerche Enrico Fermi, ITALY

## Abstract

Functional connectomics is a popular approach to investigate the neural underpinnings of developmental disorders of which attention deficit hyperactivity disorder (ADHD) is one of the most prevalent. Nonetheless, neuronal mechanisms driving the aberrant functional connectivity resulting in ADHD symptoms remain largely unclear. Whereas resting state activity reflecting intrinsic tonic background activity is only vaguely connected to behavioral effects, naturalistic neuroscience has provided means to measure phasic brain dynamics associated with overt manifestation of the symptoms. Here we collected functional magnetic resonance imaging (fMRI) data in three experimental conditions, an active virtual reality (VR) task where the participants execute goal-directed behaviors, a passive naturalistic Video Viewing task, and a standard Resting State condition. Thirty-nine children with ADHD and thirty-seven typically developing (TD) children participated in this preregistered study. Functional connectivity was examined with network-based statistics (NBS) and graph theoretical metrics. During the naturalistic VR task, the ADHD group showed weaker task performance and stronger functional connectivity than the TD group. Group differences in functional connectivity were observed in widespread brain networks: particularly subcortical areas showed hyperconnectivity in ADHD. More restricted group differences in functional connectivity were observed during the Video Viewing, and there were no group differences in functional connectivity in the Resting State condition. These observations were consistent across NBS and graph theoretical analyses, although NBS revealed more pronounced group differences. Furthermore, during the VR task and Video Viewing, functional connectivity in TD controls was associated with task performance during the measurement, while Resting State activity in TD controls was correlated with ADHD symptoms rated over six months. We conclude that overt expression of the symptoms is correlated with aberrant brain connectivity in ADHD. Furthermore, naturalistic paradigms where clinical markers can be coupled with simultaneously occurring brain activity may further increase the interpretability of psychiatric neuroimaging findings.

## Introduction

Attention deficit hyperactivity disorder (ADHD) is one of the most common developmental disorders affecting about 3-7% of children [1–3]. ADHD is characterized by inattention (e.g., being forgetful in daily activities, having trouble in organizing activities, and difficulty in following instructions), hyperactivity (e.g., restlessness and abnormally high ‘energy’ levels), and impulsivity (e.g., acting before thinking through the consequences and making hasty decisions) symptoms. Although ADHD symptom profiles are considered to be highly heterogeneous [4,5], three presentations, i.e., predominantly inattentive, primarily impusive/hyperactive and combined subtype are identified [6,7]. Irrespective of the specific characteristics, ADHD has a negative impact on many domains of everyday life, risking academic performance and causing difficulties in social relationships as well as managing home activities [8,9].

Converging evidence from different fields of research (e.g., genetics, neurobiology, neuroimaging) suggests that ADHD has a complex etiology influencing synaptic organization in the brain [10,11]. Support for this account has been found in various studies reporting alterations in functional connectivity (FC) within and between cortico-cortical (e.g., frontoparietal, dorsal/ventral attention, sensory and default-mode networks), cortico-striatal (e.g., caudate nucleus, putamen, nucleus accumbens), and cortico-limbic (e.g., cingulate cortex, amygdala, thalamus) networks in ADHD individuals [11–14]. Structural alterations are observed in largely overlapping brain areas [11,13,15], but also for instance in the brainstem [16,17]. While the early theories typically highlighted the role of a specific neuronal system such as the fronto-striatal network, the modern views acknowledge that aberrant FC in ADHD is observed in large-scale brain networks [4,18,19]. However, due to limitations of the study paradigms, inconsistencies between the experimental procedures, and heterogeneity of the ADHD populations the observed findings have been highly inconsistent across different studies [12,20]. For example, in their recent meta-analysis, Liu and colleagues [21] identified that hyper- hypoconnectivity profiles as well as specific topological configurations of the ADHD-related FC alterations were strongly age-dependent, younger children showing cortico-cortical hyperconnectivity and cortico-subcortical (i.e., putamen, pallidus, amygdala) hypoconnectivity while older children and adults showed exclusively cortico-cortical hyperconnectivity. Also within the age group the results may vary depending on the characteristics of the sample, as inattention and hyperactivity/impulsivity could be associated with partially different FC patterns [22,23].

With functional magnetic resonance imaging (fMRI), FC can be examined during different tasks or when the participants are at rest. Tonic brain activity often associated with resting state refers to the ongoing, baseline level of neural activity that is relatively stable over time. In contrast, phasic brain activity is characterized by transient, rapid bursts of neural activity that occur in response to specific stimuli or events [24,25]. Previous studies have identified various brain networks reflecting intrinsic connectivity or ’tonic background activity’ that explains the vast majority of the overall fMRI signal changes [24,25]. Some studies have reported correlations between intrinsic FC and severity of the ADHD symptoms. For instance, Mostert and colleagues found that the symptom severity was associated with stronger FC in the executive control network (specifically, in the anterior cingulate gyrus) and the cerebellar network [26]; Choi and colleagues reported that decreased FC between saliency network and sensorimotor network was associated with inattention and hyperactivity symptoms [27]; abnormal connectivity in the amygdala was shown to be associated with emotional liability symptoms in children with ADHD [28]. However, some studies do not observe such correlations [e.g., 29], and the link between FC and ADHD symptoms has remained elusive as the behavioral data reflecting the symptoms is acquired by subjectively screening everyday problems typically over the past six months and brain activity is measured when the person is relaxed in the scanner in a situation where no signs of the symptoms are present. In other words, intrinsic FC detected by the resting state fMRI (rs-fMRI) is not directly connected with symptoms expression and the studies also lack simultaneously collected behavioral markers for explaining the ongoing brain dynamics [30,31,32]. In the end, ADHD symptoms are triggered in interaction with the environment and manifest as complex goal-directed behaviors. An experimental task that efficiently provokes the complex symptoms to manifest in a particular way could further constrain the inter-individual variability in the recorded neural processes that is a key challenge in neuroimaging of developmental disorders [11,12].

Naturalistic neuroscience is an experimental approach that has recently challenged the ‘gold standard’ rs-fMRI in clinical cognitive neuroscience [11,30,33]. The aim of naturalistic neuroscience is to probe brain activity in lifelike conditions targeting the phasic brain dynamics triggered by the complex human-environment interaction [34,35]. An engaging naturalistic stimulus effectively synchronizes the underlying brain activity across participants [36], increasing the reliability [37] as well as strength [20,32] of the brain responses. Indeed, naturalistic paradigms have advantage compared to rs-fMRI in predicting cognitive and emotional characteristics of the participants [33,38,39] and the information gain can be attributed to an active task eliciting stronger connectivity [31]. Task-driven reorganization of functional connectome in ADHD mostly encompasses the fronto-parietal networks [40,41], but also altered FC in subcortical structures have been reported [42]. Altogether, task-dependent and task-independent alterations in FC may reflect different aspects in aberrant brain activity associated with ADHD [43].

Naturalistic neuroscience studies employing video viewing approach have shown promising results in detecting aberrant brain functioning in affective disorders [44,45], psychosis [46,47], autism [48–51], and ADHD [52–54]. In individuals with ADHD inter-individual synchronization of regional activity and inter-subject functional correlation are reduced compared to neurotypical controls during viewing a naturalistic stimulus in several brain areas, including the anterior cingulate cortex, supratemporal, occipital, and posterior parietal regions [52,53]. Stronger inter-individual synchronization of brain activity in the ADHD *vs.* control group, in turn, has been observed in the occipital and temporal lobes, as well as in the amygdala and insular cortex [52]. Recent studies [55,56] have further examined FC in children with ADHD and controls in naturalistic tasks. Fisher and colleagues [55], for instance, observed that cognitive and perceptual load differently affect brain network efficiency in a naturalistic task. Pho and colleagues [56], in turn, reported developmental trajectories of FC patterns during video viewing in children from age 6 to 16, demonstrating that age and cognitive abilities differentially reflect to FC in children with ADHD and in controls. Although video viewing limits spontaneous expression of symptoms and provides rather restricted behavioral data [57], these studies were able to explain inter-individual variability in the brain activity associated with subjectively evaluated ADHD symptoms collected with a screener.

Virtual reality fMRI (VR-fMRI) potentially circumvents many of the limitations of the video viewing studies [30,58]. While the dynamic and rich video stimuli reach featural complexity and dynamics of naturalistic situations, this paradigm lacks a fundamental aspect of everyday experience – the possibility to interact with the environment [59]. Unlike passive observation of videos, VR paradigms enable execution of volitional behaviors that can be operationalized to carry information reflecting, for instance, attention, memory, decision making, reacting to rewards, getting distracted, and expressing hyperactivity. Successful implementation of goal-directed behavior in everyday life involves orchestrating various cognitive processes simultaneously. This complexity is not well represented in traditional task-based neuroscience paradigms that typically focus on targeting an isolated cognitive construct. VR allows to emulate complex scenarios offering researchers opportunities to investigate human cognition and behavior in a controlled yet naturalistic interactive setting. However, VR-fMRI has not been utilized in studying neural underpinnings of ADHD so far.

Here we propose a novel naturalistic task for VR-fMRI that allows precise quantification of rich goal-directed behaviors during execution of open-ended tasks [60,61,62]. In this Executive Performance in Everyday Living (EPELI) game, participants perform everyday chores in a virtual apartment, in life-like situations corresponding to those where ADHD symptoms typically manifest [63,64,65]. Indeed, the game provides an excellent accuracy for detecting individuals with ADHD based on game-related behavior (88%) [63] or eye movements (92%) [64]. ADHD symptoms can be observed in EPELI, for example, as forgetfulness to do the instructed tasks, committing more irrelevant actions per successfully conducted tasks, poorly organized navigation in the environment, and conducting higher number of impulsive actions [63,64].

We studied FC in children with ADHD and typically developing (TD) controls during EPELI VR game playing and two widely used fMRI paradigms (video viewing, and rs-fMRI). To control for the contextual differences between active task execution in the EPELI game and passive video viewing, we selected screen recordings of EPELI game play for the video viewing condition. In the data analysis, we first employed network-based statistics (NBS) that is designed to detect subnetworks (several links forming a coherent ‘connected component’) differentiating between the groups or conditions in a data-driven manner [66]. We also tested group differences in FC on link-by-link basis in case not a single connected component was detected by NBS [66]. The advantage in these two approaches is that they require minimal a priori assumptions on size or spatial distribution of the FC networks [66], which is crucial in brain imaging of ADHD as there is no established topological architecture for the related reorganization in FC [11,12,20]. In previous studies, these two methods have revealed decreased FC in ADHD during resting state, in particular, in the fronto-occipital [67] and fronto-parieto-cerebellar networks [68], as well as during executive task performance in the parieto-temporo-occipital network [69]. However, there is also evidence of hyper-connectivity in ADHD in specific networks (e.g., amygdalar, prefrontal and temporo-occipital networks) [70,71].

In addition to NBS and link-wise analysis, we ran graph theoretical analysis focusing on integration and segregation of FC networks [72]. Previous studies employing graph metrics have revealed altered global and local FC network topology in individuals with ADHD [73–78]. In the present study, four graph metrics were selected to examine network integration and segregation properties. (1) Global efficiency, reflecting how efficiently information can be transferred across the entire network, appears to be reduced in ADHD population [73–75]. (2) Betweenness centrality, showing how important a specific node is in the transmission of information across the network, in turn, is one of the graph metrics with relatively high predictive power for classification of ADHD cases [76]. (3) Participation coefficient, capturing degree of integration across subnetworks, is increased in ADHD [77]. Finally, (4) node degree indicating the contribution of different regions into network integration (i.e., a higher nodal degree means that the node has more connections with other nodes in the network) has been reported to be lower in ADHD on average [78], while in specific networks it can be either decreased (e.g., visual, dorsal attention and default-mode networks [79–81]) or increased (e.g., limbic and saliency networks) [79–81].

Our preregistered (https://tinyurl.com/4eujtt42) hypotheses were: (1) We will observe both paradigm-general as well as paradigm-specific FC patterns separating ADHD participants from TD controls [43]. Paradigm-general effects were expected as intrinsic FC should be reflected to each experimental condition in a largely similar manner [82–84]. Paradigm-specific effects, in turn, were expected to be observed in brain networks associated with atypical goal-directed behavior (e.g., fronto-parietal, fronto-temporal, and fronto-striatal networks) during EPELI gameplay that provokes symptoms expression, while during video viewing aberrant FC would be restricted to the dorsal attention and sensory networks [52,53]. Such findings delineating group differences in FC patterns associated with intrinsic processes and two types of naturalistic conditions, passive viewing of a naturalistic stimulus and active exploration in virtual environment, would provide novel evidence for the role of overt expression of symptoms and human-environment interaction in driving aberrant FC in ADHD. (2) Inter-individual FC variability explaining the symptoms was hypothesized to be higher during EPELI performance than during resting state or passive video viewing. In other words, measuring the ongoing symptoms with precise quantitative means and simultaneously recording phasic brain dynamics was expected to allow more precise FC-to-symptom mapping than conventional rs-fMRI analysis, where tonic states in the intrinsic brain connectivity are linked to subjective reports reflecting manifestation of symptoms over periods of several months, or more restricted naturalistic paradigm.

## Materials and methods

### Participants

Thirty-nine ADHD and 37 TD children took part in the study between the 15^th^ of December 2021 and the 2^nd^ of April 2023 (see Participants section in S1 Appendix in S1 File). The Helsinki University Central Hospital Regional Committee on Medical Research Ethics approved the research protocol. All participants and their guardians gave their written informed consent according to the Declaration of Helsinki.

Inclusion and exclusion criteria followed the previous studies [63,64]. All the participants in both groups were Finnish native speakers. The participants were accepted to the TD group if they did not have any diagnosis related to psychiatric (diagnosis F00-F00 in ICD-10) or neurological (G00-G99 in ICD-10) conditions and were not entitled to special support at school. Participants were potentially eligible for the clinical group if they had ADHD diagnosis (F90) made by a licensed medical doctor, which was verified via the National Medical Database (see S1 Appendix in S1 File). For screening the ADHD symptoms and other psychiatric symptoms we used ADHD Rating Scale IV (ADHD-RS) [85] and Child Behavior Check List (CBCL) [86]. In both scales, higher scores refer to more prominent symptoms. Participants with ADHD were asked to take 24 hours break from their stimulant medication before coming to the study, except one participant who was taking guanfacine that has a long-lasting effect.

Data of some participants was excluded from the analysis due to lack of data, comorbid diagnosis (see S1 Appendix in S1 File) or excessive movements during the measurement (see Data preprocessing). The final sample included 31 ADHD and 34 TD participants. The groups did not differ significantly in age, gender, handedness, or general abilities (see Table 1). As expected, the ADHD group had more psychiatric symptoms than the TD group, indicated by the ADHD-RS and CBCL scores (Table 1). Moreover, none of the TD participants scored on ADHD-RS above 85th percentile on ADHD-RS, indicating that the probability of any of the participants in the control group had an undiagnosed ADHD was low. In addition to that, none of the controls scored in the clinical range on CBCL total score [87], indicating low probability of other undiagnosed mental health conditions in the TD group.

**Table 1:** Background characteristics of the sample.

	ADHD	TD	Statistical test
**Gender**	20 boys, 11 girls	19 boys, 15 girls	X^2^ = 0.367, p = 0.61
**Age**	11.47 (1.28)	11.82 (1.07)	t(54.77) = 1.15, p = 0.26
**Left-handed**	n = 2	n = 1	–
**WISC-IV**	10.02 (2.59)	11.33 (2.12)	t(44.21) = -1.99, p = 0.053
**ADHD-RS (Total score)**	31.33 (11.67)	6.06 (4.02)	t(35.06) = 11.28, p < 0.001
**ADHD-RS (Inattention subscale)**	17.37 (5.88)	3.88 (2.80)	t(40.37) = 11.46, p < 0.001
**ADHD-RS (Hyperactivity-impulsivity subscale)**	13.97 (6.74)	2.17 (2.07)	t(33.80) = 9.20, p < 0.001
**CBCL**	46.2 (21.89)	14. 39 (10.10)	t(39.95) = 7.28, p < 0.001

WISC-IV =  Wechsler’s Intelligence Scale for Children [88], averaged scores from two subtests (Vocabulary and Similarities); ADHD-RS =  ADHD Rating Scale-IV, parent form [85]; CBCL =  Child Behavior Checklist [86].

### Experimental conditions

EPELI [63] is a VR game designed to quantify execution of goal-directed behavior [60–65] and has been validated as a tool for detecting ADHD in children [60,63,64]. The MRI version of EPELI running on a desktop computer [60] and projected via mirror to a screen in the scanner includes ten task scenarios, plus a practice scenario at the beginning of the gameplay when the scanning had started. At the beginning of each task scenario, participants were presented with audio instructions for the current scenario, after which they performed the given tasks. Navigation and interaction with game objects were carried out with an MRI-compatible trackball. The sound was delivered with Sensimetrics S14 insert earphones. The total duration of the 10 EPELI scenarios was on average 23 ± 1.4 minutes. The Unity game engine running EPELI game stores a detailed log file of the game play. Purpose-built scripts were used to extract the previously validated performance measures from this data containing timing and content of any game interaction and movement of the participant (see [63,64] for more details). The main EPELI behavioral outcome measures were: *Total Score* (percentage of correctly performed subtasks), *Task Efficacy* (percentage of relevant actions out of all actions), *Navigation Efficacy* (Total Score divided by distance covered), and *Total Actions* (number of track ball controller clicks). These measures reflect problems typical for children with ADHD [63,64]. Lower Total Score reflects poorer ability to memorize and execute multistage tasks in the naturalistic setting, lower Task Efficacy suggests weaker selective attentional and executive control, lower Navigation Efficacy indicates problems with planning and navigation, and higher number of Total Actions is presumed to be associated with impulsive behavior [see 63 and S1 Appendix in S1 File for more detail]. Out of these measures, especially Task Efficacy is shown to be sensitive in discriminating between participants with ADHD from TD controls, with ADHD participants showing lower Task Efficacy.

In the Video Viewing task, participants were presented with four video fragments of EPELI gameplay [64]. Two video fragments were from gameplay of participants with ADHD, and two from the TD group gameplay recordings (see https://youtu.be/oo2LMhShKJM for a video). Each fragment had about two-minute duration and was followed by questions related to the video (see S1 Appendix in S1 File for more detail).

During the resting state recording the participant was instructed to stay still and look at any corner of the square presented at the center of the screen. The participants were instructed to shift their gaze to a different corner when they feel like it to keep themselves focused and relax their eyes. The duration of the resting state scan was eight minutes.

### Procedure

The MRI measurements were conducted in the following order: EPELI; Video Viewing; structural MRI; Resting State. The order of the tasks was defined based on pilot measurements: the most engaging condition, the VR game, was administrated first to minimize stress and discomfort of the participants during the scan and ensure data quality and participant cooperation. After scanning the screening instruments, questionnaires, and tasks were administrated (see S1 Appendix in S1 File).

### MRI data acquisition

Participants were scanned using a 3 Tesla MAGNETOM Skyra whole-body scanner (Siemens Healthcare, Erlangen, Germany), using a 30-channel head coil at Advanced Magnetic Imaging Centre (Aalto University).

A T1-weighted Magnetization-Prepared Rapid Acquisition with Gradient Echo (MPRAGE) 3-D image was collected for each participant. For the registration, T1 images of the participants were resampled to 3 mm.

Blood Oxygenation Level Dependent (BOLD)-weighted functional imaging data were collected with Simultaneous Multi-Slice sequence using T2 * -weighted echo-planar imaging (TR =  594 ms, TE =  16 ms, flip angle =  50°, field-of-view read =  192 mm, field-of-view phase =  100%, 44 slices covering the whole brain, phase encoding direction anterior to posterior, isotropic 3 mm voxels). More details on the brain imaging in S1 Appendix in S1 File.

### Data preprocessing

Preprocessing was done with fMRIPrep toolbox (https://fmriprep.org) [89,90]. FreeSurfer (https://surfer.nmr.mgh.harvard.edu/) [91] was called to reconstruct surfaces from T1-weighted structural images, and all fMRI data was registered to a pediatric template (MNIPediatricAsym:cohort-5) [92,93].

Denoising included detrending of the signal and confounds (4 head motion parameters and 8 cerebrospinal fluid and white matter parameters) and regressing out the confounds. After that the signal temporally filtered and demeaned. The high-pass filter (cut-off 0.01 Hz) was selected for task-based analyses instead of band-pass filtering as the low-pass filter could discard meaningful signals and rapid MRI sequence used in the data acquisition was shown to be less affected by physiological noise [94]. For Resting State data, we chose the common band-pass filter (0.01-0.1 Hz) settings.

To minimize the possible effects of motion artifacts, volumes contaminated by large movements (frame-wise displacement, FD >  0.5 mm) were removed prior to computing FC. If any of the three scans had more than 30% of the total volumes removed with these criteria it was excluded from the analysis (see S1 Appendix in S1 File). That resulted in 26 ADHD and 33 TD participants with EPELI, 30 ADHD, and 33 TD participants with Video Viewing task, and 28 ADHD, 32 TD participants with Resting State scan. After the removal of the affected volumes, the groups did not differ in the mean FD of the kept volumes in any of the three paradigms (see Data Preprocessing section in S1 Appendix in S1 File for more detail).

### Data analysis

Two hundred ten cortical and thirty-six subcortical subregions were extracted based on Brainnetome Atlas (http://atlas.brainnetome.org/index.html) [95] with Nilearn python package v 0.10.0. Average data of each subregion was also used to calculate the connectivity matrices with the same package. Percentage of removed volumes and mean FD of the kept volumes were regressed from the connectivity matrices to ensure that the group differences are not inflated by higher movement rate in the ADHD group.

NBS comparing the groups and the paradigms was conducted with a related MATLAB toolbox (https://www.nitrc.org/projects/nbs/) [66]. The statistical testing in NBS that applies topological clustering algorithm and family-wise error correction was run with 5000 permutations with primary thresholds t =  3.5 (group comparisons) and t =  4.0 (linear regression analyses, for the results with alternative thresholds see S1 and S2 Figs in S1 File). In addition, we assessed a general effect of task performance on the group differences by regressing Task Efficacy from the EPELI FC data. To test associations between FC and task performance as reflected in Task Efficacy measure, NBS analysis employing linear regression was run separately for each group, by including age and gender as covariates. The covariates were added to the linear regression analysis as Task Efficacy as well as symptom profiles are associated with age and gender [62]. Similar linear regression analysis was conducted to test the association between FC and behavioral problems assessed via screening questionnaires (ADHD-RS and CBCL). In the models testing for group differences, covariates were not used because the groups were balanced in terms of age and gender.

Finally, similar group comparisons and linear regression analyses than described above were also conducted with link-based testing controlled by the false discovery rate (FDR) [66,96], in case significant effects in NBS were not observed. This procedure was applied to test if there is any difference in FC at the level of isolated links, which is not detected by NBS method [66]. The FDR statistics were computed with the NBS toolbox with 50 000 permutations.

To examine task-general FC effects, NBS was run on normalized connectivity matrices averaged across the three paradigms for each participant. A supplementary analysis for the main effect of Condition was conducted with NBS ANOVA to confirm that the lack of task-general effect is not due to averaged FC matrices [97]. This analysis was applied to the participants who had data in all the three conditions (23 ADHD and 31 TD).

Graph theoretical analysis was implemented with Brain Connectivity Toolbox for MATLAB [98]. Prior to the analysis, all negative connections were zeroed out, and the connectivity matrices were thresholded individually for each participant to keep 10% of connections. In the graph theoretical analysis with multiple layers (EPELI, Video Viewing, Resting State), participant’s FC in each paradigm was modelled with Pymnet Python package [99]. In this analysis, a node represented a single brain parcel, intra-layer links represented FC between the regions in a specific experimental condition. Assessed measures were similarity between the layers calculated as nodal degree overlap and local clustering coefficient [100] calculated at each node and then averaged. For calculation of these metrics, the connectivity matrices were thresholded to keep 10% of connections for each participant. Nodal degree overlap was calculated as how many times each edge appears in exactly that subset for every non-empty subset of layers. As an additional measure of similarity between layers, we calculated correlation between two corresponding links in each layer.

Statistical significance in the graph theoretical analysis was assessed by permutation test following by FDR multiple comparisons correction [96]. The same correction was applied in the analysis of behavioral data. To examine the correlation between ADHD-RS and the EPELI measures we used Kendall’s rank correlation as ADHD-RS distribution was skewed due to low symptoms score in the TD group.

## Results

### Behavioral results

The ADHD group demonstrated worse performance in EPELI compared to the TD group, with lower Total Score (t(54) =  3.53, p <  0.001) and lower Task Efficacy (t(57) =  2.64, p =  0.011). Navigation Efficacy (t =  1.51, df =  53.72, p =  0.14) and Total Actions (t(56) =  1.33, p =  0.19) did not differ between the groups.

ADHD-RS scores across all participants were correlated with Total Score (τ =  -0.30, p <  0.001), Task Efficacy (τ =  -0.26, p =  0.0038) and Total Actions (τ =  0.21, p =  0.021), but not with the Navigation Efficacy (p =  0.052).

Based on the follow-up questions, participants with ADHD experienced the videos as more difficult to follow than TD participants (t(62) =  4.21, p <  0.001). Like EPELI measures, the ratings of the difficulty to follow the videos were correlated with ADHD-RS scores across all participants (τ =  0.24, p =  0.0077). There were no group differences in the ratings on how interesting the video was (t(57) =  0.80, p =  0.43), nor how well the participant could focus on it (t(63) =  1.58, p =  0.12), and these ratings were not correlated with the ADHD-RS scores either (τ =  -0.090 p =  0.31, and τ =  0.15, p =  0.087).

### FC during EPELI

#### NBS.

**Fig 1:**
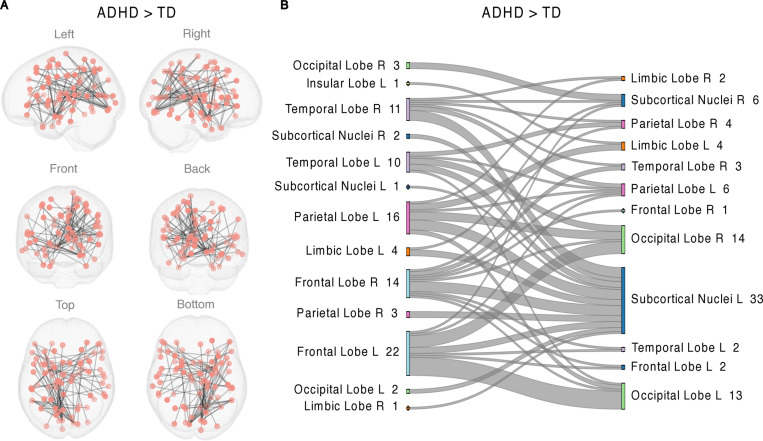
A network revealed by NBS showing stronger FC in the ADHD group than in the TD group during EPELI gameplay. No differences were observed in the opposite direction. The primary statistic threshold in these analyses was **t** =  3.5. (A) anatomical representation, (B) alluvial diagram. **L** =  left; **R** =  right; a number indicates the count of significant links related to the area. Both columns represent the same contrast (ADHD>TD) and were assigned to the areas for visualization purposes.

NBS revealed a large connected component with 77 nodes and 90 edges differentiating FC in the ADHD and TD group during the EPELI gameplay (Fig 1, Table 2). As illustrated in an alluvial diagram, increased FC in the ADHD compared to the TD groups was most prominent in the subcortical nuclei, but observed also in the frontal and occipital areas (Fig 1, panel B). FC during EPELI in the two groups separately can be found in supporting information (S3 Fig in S1 File). The analysis that regressed general task performance effect out from EPELI FC data revealed one component with group differences in 108 nodes and 152 edges, indicating hyperconnectivity in the ADHD group. Majority of the nodes in this component were located in the frontal and subcortical areas (see S4 Fig, and S3 Table in S1 File).

**Table 2:** NBS analysis of EPELI FC contrasting ADHD and TD groups. The table shows the nodes with the highest number of significant connections in the network detected by the NBS analysis of EPELI FC contrasting ADHD and TD groups.

Node Degree	Area^1^	Region^1^	Network^2^
12	Lateral Amygdala, L	Subcortical Nuclei	Subcortical
12	Caudal Lingual Gyrus, R	Occipital Lobe	Undefined
11	Rostral Cuneus Gyrus, L	Occipital Lobe	Visual
6	Caudal Posterior Superior Temporal Sulcus, L	Temporal Lobe	Ventral Attention
6	Caudal Area 7, L	Parietal Lobe	Dorsal Attention
6	Posterior Parietal Thalamus, L	Thalamus	Subcortical

For the full list of significant nodes see S2 Table in S1 File.

L = left; R = Right.

1 - Based on Brainnetome Atlas [95].

2 - The network is assigned as defined by Power and colleagues [101] by closest centroid

**Fig 2:**
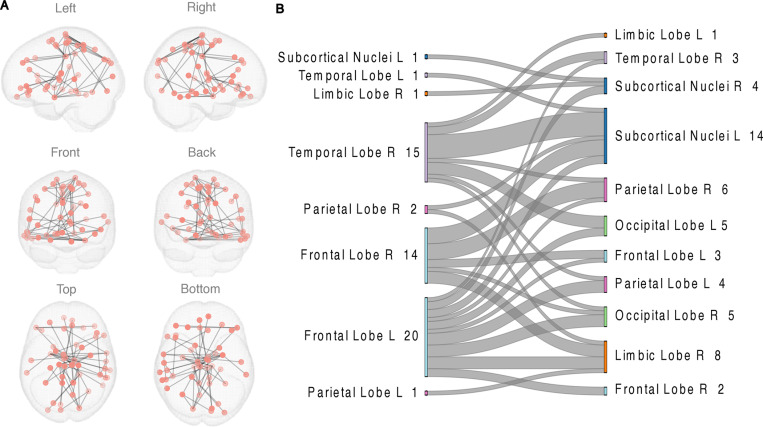
The network revealed by NBS that was positively associated with Task Efficacy in EPELI game in the TD group. Age and gender were included as covariates. The primary statistic threshold in these analyses was **t** =  4. (A) anatomical representation, (B) alluvial diagram. **L** =  left; **R** =  right; a number indicates the count of significant links related to the brain area. Both columns represent the same contrast and were assigned to the areas for visualization purposes.

FC during EPELI gameplay was positively associated with Task Efficacy in the TD, but not in the ADHD group. The network showing correlation between FC and Task Efficacy in TD group consisted of 50 nodes and 55 edges (Fig 2, S4 Table in S1 File). Although 21 of these nodes were overlapping with the network detected in the group comparison (see S5 Fig in S1 File), this network clearly reflected functional synchronization between different brain structures, as the networks shared only one common edge (between Inferior Frontal Gyrus and Amygdala). No associations between FC during EPELI gameplay and other main EPELI performance measures were found, nor with FC during EPELI gameplay and ADHD-RS or CBCL scores.

#### Graph theory

There was a group difference in the node degree in one node located in the left inferior temporal gyrus for FC obtained during EPELI task, with the ADHD group having a higher node degree than the TD group (see S6 Fig in S1 File). There were no group differences in other graph metrics for FC during the EPELI gameplay.

### FC during Video Viewing task

#### NBS.

For Video Viewing task, NBS analysis did not indicate any significant differences between the groups (see S7 Fig in S1 File for FC patterns during video viewing). However, there was a positive association between FC during Video Viewing task and Task Efficacy score in EPELI in the TD group (Fig 4), which was not observed in the ADHD group. Similarly, there was a negative association between CBCL and Video Viewing FC observed only in the TD group (Fig 5). No association between FC during the Video Viewing task and ADHD-RS scores was found.

**Fig 3:**
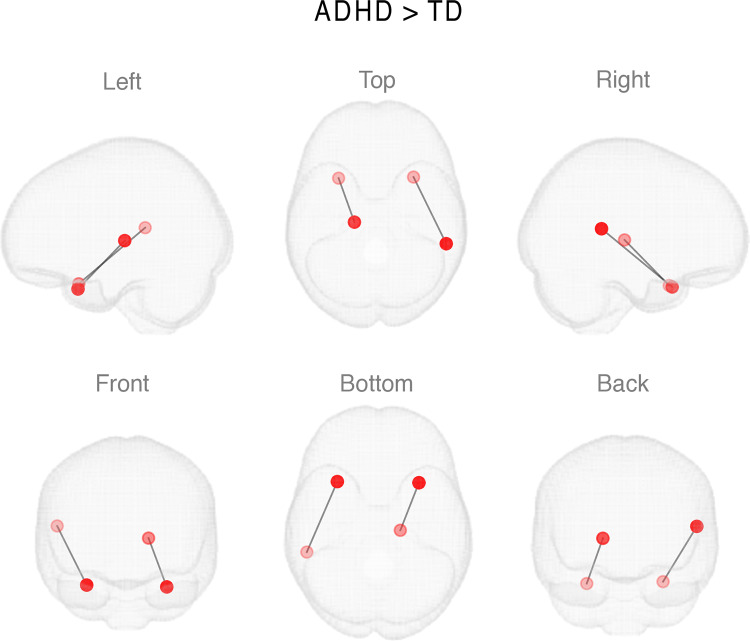
The results of the link-based analysis for FC differences between the groups during Video Viewing task.

**Fig 4:**
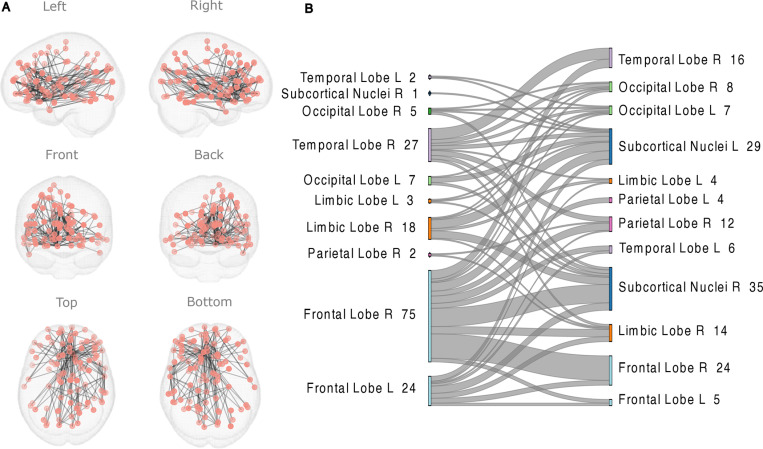
The FC network in Video Viewing detected by NBS that was positively associated with Task Efficacy in EPELI in the TD group. The NBS results were obtained with the primary statistics threshold of t = 4. Age and gender were included as covariates.

**Fig 5:**
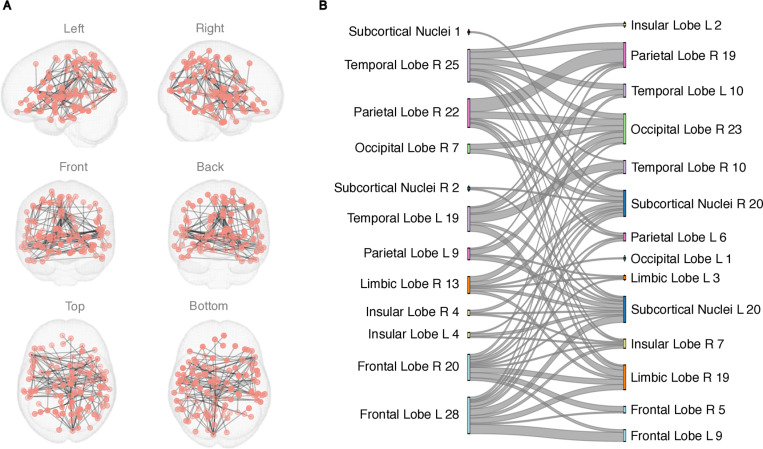
The FC network in Video Viewing detected by NBS that was negatively associated with CBCL score in the TD group. Age and gender were included as covariates. The primary statistic threshold in this analysis was **t** =  4. (A) anatomical representation, (B) alluvial diagram. **L** =  left; **R** =  right; a number indicates the count of significant links related to the brain area.

Link-wise analysis with FDR controlled statistics revealed two connections that were stronger in the ADHD group compared to TD group in the Video Viewing task. As shown in Fig 3, these connections were in temporo-thalamic (between the left superior temporal gyrus and left thalamus) and intra-temporal (the right superior temporal gyrus and right posterior superior temporal sulcus) networks.

#### Graph theory

During Video Viewing there were no differences between the groups in any of the graph metrics.

### FC during the resting state

#### NBS.

**Fig 6:**
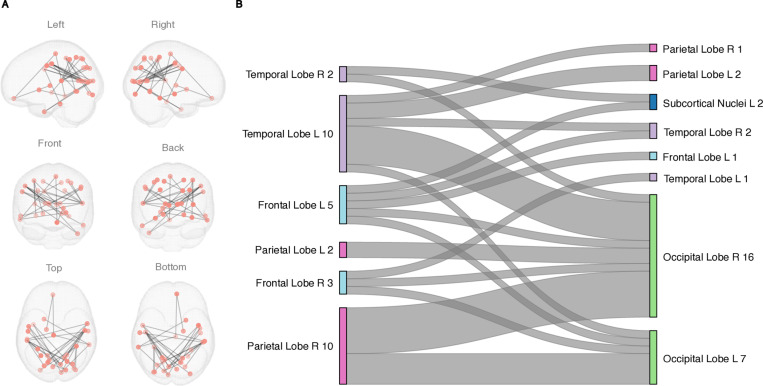
The network revealed by NBS method that was negatively associated with ADHD-RS in the TD group. Age and gender were included as covariates. The primary statistic threshold was **t** =  4. (A) anatomical representation, (B) alluvial diagram. **L** =  left; **R** =  right; a number indicates the count of significant links related to the brain area.

**Fig 7:**
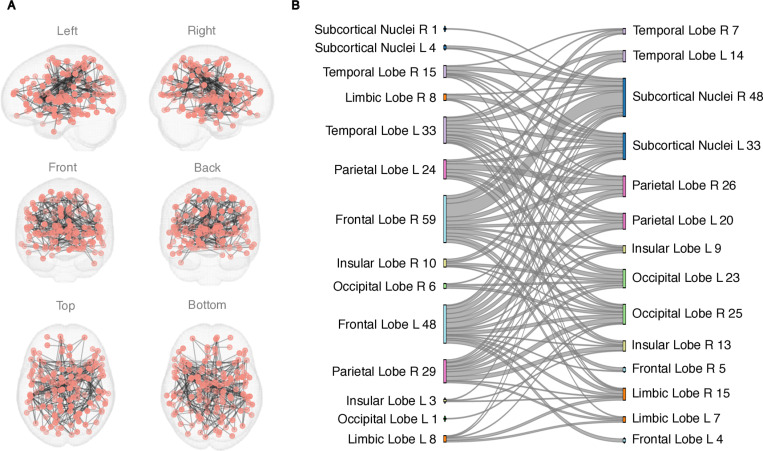
The FC network in Resting State detected by NBS that was negatively associated with CBCL score in the TD group. Age and gender were included as covariates. The primary statistic threshold in these analyses was **t** =  4. (A) anatomical representation, (B) alluvial diagram. **L** =  left; **R** =  right; a number indicates the count of significant links related to the brain area.

In Resting State condition, no group differences in FC were observed either with NBS or with link-wise analysis (FC patterns during Resting State can be found in S8 Fig in S1 File). However, there was a negative correlation between Resting State FC and questionnaire data both for ADHD-RS and CBCL scores in the TD group (Fig 6 and 7). No association between EPELI performance measures was found in the data collected in Resting State condition.

#### Graph theory

There were no differences between the groups in any of the graph theoretical metrics in Resting State condition.

### Similarities between the three fMRI conditions

#### NBS.

According to NBS analysis, there were no group differences in the connectivity matrices averaged across the three scans. The link-wise analysis, however, detected one connection that showed group differences regardless of the condition. This link connects the right inferior frontal gyrus and the left superior temporal gyrus. NBS ANOVA analysis did not indicate significant effect of Condition.

#### Graph theory.

There were no group differences in FC patterns shared across the three conditions based on the three-layer multiplex network analysis.

## Discussion

The goal of the present study was to examine how aberrant FC in individuals with ADHD manifests in a situation where overt symptoms are present and in two other conditions, one lacking execution of goal-directed behavior but matching with the VR task in stimulus contents, and the other specifically capturing intrinsic activity. Moreover, with correlations between objective and subjective symptom measures and brain activity, we investigated how the measured FC patterns in these three experimental conditions are coupled with behavioral measures. As our first preregistered hypothesis predicted, there was a significant difference in FC patterns between the ADHD and TD groups during the VR task. Subtraction of task performance effect resulted in largely overlapping pattern showing group differences in the same networks containing various cortical and subcortical areas (especially pronounced in frontal, occipital, parietal and striatal areas with highest nodal degree in the medioventral occipital cortex, thalamus, inferior frontal gyrus, and superior/inferior parietal cortex), suggesting that these areas are involved in ADHD even when the cognitive performance is considered. Although group differences in FC at link-level were detectable also in the passive viewing condition, they involved a much more restricted brain network compared with those highlighted during active execution of goal-directed behavior. These findings are consistent with those of Fisher and colleagues [55], which showed that FC effects of ADHD are dependent on the cognitive load. Also, the paradigm-general group differences in FC assumed to raise from intrinsic activity were rather limited. One factor that could explain the lack of group differences in resting state data and limited paradigm-general effects, is that different symptom domains show correlation with specific brain activity patterns [22,23,102], which could not be separately examined in this sample.

We found partial support to the second preregistered hypothesis predicting that measuring active goal-directed behavior in VR may allow more precise FC-to-symptom mapping compared with conventional rs-fMRI analysis where intrinsic brain connectivity is being associated with subjective symptom reports, however, the effect was observed only in the TD group. In TD participants, FC during both active VR-gameplay as well as gameplay viewing were correlated with behavior, but only with the performance in the naturalistic EPELI task collected in the scanner. In both fMRI conditions Task Efficacy scores correlated with FC in a large-scale brain network encompassing mainly frontal, temporal, limbic and striatal areas. At the same time, Resting State FC in the TD participants was associated with parent rated ADHD symptoms, but not with objective behavioral measures. Different topography in coupling of phasic activity (VR-gameplay, gameplay viewing) and objective behavioral measures on one side and tonic activity (Resting state) and parent ratings of the symptoms on the other side emphasize different brain correlates underlying the observed phenomena. Altogether, the observed results highlight the opportunities of active naturalistic tasks in studying aberrant brain functioning in developmental disorders such as ADHD, but also provide evidence of the possible complementary benefits for studying attention between different experimental approaches. For understanding the similarities and differences between the tonic activations induced by the VR-gameplay and gameplay viewing, more research is still needed.

Interestingly, the network revealed by the group comparison showed hyperconnectivity associated with ADHD while the correlational analysis in TD participants revealed a network where higher FC was associated with higher efficacy. Similarly, Kucyi and colleagues [103] observed that the relationship between inattention and individual differences in FC was reversed in the ADHD and TD groups. It is important to note that the networks revealed by the group comparison and the correlational analysis in our study had largely different spatial configurations, and they may hence serve different brain mechanisms. Consistent with our findings, there are also other studies showing brain network alterations associated with attention among the typical developing population [43,104,105]. It is possible that ADHD involves regulatory mechanisms that differ from those seen in typically developing individuals [106], and that the relationship between FC and symptoms may not follow a simple linear pattern.

It is also noteworthy that previous studies commonly report both hyperconnectivity and hypoconnectivity in ADHD, in distinct brain networks [18,29,107]. As FC in the TD group was negatively associated with CBCL or ADHD-RS scores and positively associated with Task Efficacy, it might be considered as hypoconnectivity in relation to ADHD symptoms, in line with previous studies [107–110]. Such FC pattern was not observed in the ADHD group, which could be due to heterogeneity of the ADHD group that is widely considered challenge in neuroimaging of ADHD [4,5,11]. This interpretation is supported by findings that attention-related brain network alterations similar to observed in ADHD also exist among the TD population [43,104,105], which aligns with dimensional perspective on ADHD [106]. However, further research is needed to determine which of the two suggested interpretations is most accurate.

Whereas numerous rs-fMRI and task-based fMRI studies with restricted tasks have examined the neural basis of ADHD [12,111], studies employing naturalistic paradigms where the individuals actively take part into situations where their problems occur have been lacking. The findings related to the comparison of rs-fMRI and the two ecologically relevant paradigms in the present study clearly reveals the complementary benefits of naturalistic designs: the clinical group displayed no differences from typically developed peers in tonic background activity during resting state but showed clear alterations in phasic brain connectivity triggered by the execution of goal-directed behaviors in cortico-subcortical (e.g., cortico-striatal and cortico-limbic) and cortico-cortical (e.g., fronto-temporal) networks.

Although there were no robust differences among the three conditions, S3, S7, and S8 Figs in S1 File illustrate clear changes in the FC patterns related to the experimental manipulation. While we expected group differences also in the intrinsic connectivity, the limited findings on this domain cannot be considered totally unexpected as even the most robust group differences, such as alterations in the DMN and executive control network, are not replicable across the datasets [112]. The striking influence of an active naturalistic task on aberrant FC in ADHD is a novel finding. It is noteworthy, however, that several previous studies have demonstrated in TD participants that performance of an active task, even a simple attention test, considerably changes the FC patterns in the brain compared with resting state condition [40–42] and such effects are also reported in ADHD [43]. Together with important findings obtained with video viewing [57], these advancements in ecologically relevant experimental approaches advocate for a major paradigm shift in clinical cognitive neuroscience.

The observed group differences in FC during the VR task were widely distributed across cortico-striatal, cortico-limbic, and cortico-cortical networks, both in the direct group comparison and in the analysis adjusted for task performance effect. While large-scale group differences between participants with ADHD and TD controls have been observed also in previous studies with NBS both in children [68,74] and in adults [70,113], the somewhat unexpected finding that the network covered additional areas besides those typically activated in tasks targeting executive control [19,114] may have important implications. Such global changes in FC are in agreement with modern accounts describing the neural underpinnings of ADHD [4,13] and also logically follow from studies demonstrating the widespread nature of attention-related modulations in the human brain. For instance, Gonzales-Castillo and colleagues [115] demonstrated that even a simple attention task activates over 95% of the cerebral cortex. In this light, the current cognitive neuroscience literature highlighting the role of particular brain regions in specific attention-related processes or specific disorders of attention may give an oversimplified picture [116]. The present findings imply that the focus should be transferred from identifying the static functional interpretations for individual brain areas to mapping the complex symptom patterns to phasic brain states that are dynamically influenced by human-environment interaction [30]. We presume that the observed group differences are related to active execution of the tasks in interaction with the environment in the VR condition, and are not driven by the complexity or properties of visual input itself, as the visual input in the VR task and Video Viewing conditions utilized in the study can be considered comparable (the videos were presented with the same equipment, and they illustrated scenarios where participants had played the same game). It is, however, important for future research to investigate which specific characteristics of a VR paradigm are the most relevant for provoking the differences in FC characterizing different clinical groups.

The network differentiating the ADHD and TD group involves considerable proportion of altered connections in subcortical structures. These subcortical areas, for example, in the striatum and limbic system play a key role in neuromodulation and have been implicated in ADHD in various studies [4,19,117–119]. More specifically, hyperconnectivity of the subcortical areas is a mechanism noted in multiple theories of ADHD, starting from “energetic” theories [120] to fronto-striatal models [19], and beyond [19]. For instance, the striatal structures such as the caudate nucleus and putamen are involved in manifestation of ADHD symptoms, contributing to difficulty in maintaining attention, controlling impulses and reduced executive control. Amygdala and thalamus affected by norepinephrine and dopamine levels mediate level of alertness, impulse control, and processing of emotional stimuli including reward [4,19,121,122]. Lower dopamine level in hippocampus has been associated with ADHD symptoms in animal models, and linked to learning and memory difficulties [123]. In the context of EPELI, individuals with ADHD are demonstrated to perform worse in spatial navigation [63,64], which could be linked to hippocampal function [124,125]. Overall, dysfunction of subcortical structures has been previously associated with deficits in affective and cognitive components of executive control that are widely recognized as clinically relevant problems in ADHD [4,11]. The complex interplay between subcortical nuclei (e.g., amygdala, thalamus, striatum) and various cortical regions (frontal, temporal, parietal, and occipital areas) associated with reward processing pathways, executive control processes and the alertness network [4,19] underscores the complexity of neurobiological basis of ADHD, highlighting the need for a comprehensive understanding of interaction of large-scale brain networks.

The group differences in FC during the video viewing were restricted to the temporal lobe networks along with a temporo-thalamic link. These temporal regions are overlapping with those observed in the previous video viewing study in participants with ADHD [53]. These higher-level temporal areas are considered as convergence zones supporting, for instance, parsing higher-level semantic representations [126] and integrating information from different modalities [127]. It is noteworthy, however, that the videos used in the present study were not built specifically for detecting ADHD symptoms [57], but rather selected based on stimulus information that is comparable to the VR task. Hence, we do not expect that the present findings bring out the best in various opportunities of using dynamic audiovisual stimuli with rich contents in ADHD research [53]. Nevertheless, our findings support that clearly the task requiring active execution of goal-directed behaviors elicits more widespread FC differences than a comparable passive task.

The group differences in EPELI performance and the associations between inter-individual task performance and parent-rated symptom severity were largely consistent with previous behavioral studies [63,64], corroborating the applicability of this task for objective quantification of the symptoms in parallel with acquisition of functional imaging data. Task Efficacy, reflecting executive control and calculated as proportion of relevant actions out of all actions performed by the participant, was associated with connectivity strength in large scale networks where frontal, temporal, and subcortical areas were highlighted. In FC extracted when participants played EPELI, association with the measure reflecting the amount of irrelevant actions per successfully performed task was observed mostly in the connections between temporal and subcortical regions (amygdala and thalamus), as well as frontal regions, which might be indicative of active cognitive control and efficient integration of sensory and emotional information necessary for high task performance. At the same time, during Video Viewing bigger connected component with highest links in frontal areas was associated with the same EPELI measure (Task Efficacy), this could reflect low-effort engagement with the content of the videos for the participants with higher performance in the EPELI task. These differences in FC-to-symptom correlations between EPELI and Video Viewing could either reflect distinct task demands or just different associations with the performance. In both cases, those participants that were less effective in focusing on the relevant actions in the gameplay showed stronger FC. Because this association was not observed in tonic background activity, as higher FC was associated with lower symptoms, we assume that task-related hypoconnectivity during EPELI and Video Viewing could be related to inefficient brain network functioning, with insufficient allocation of the resources or insufficient synchronization of the brain areas. CBCL, in turn, was negatively associated with FC in Video Viewing and Resting State in TD group, but not in EPELI, which could indicate that EPELI is specifically evoking brain activity characterizing attention regulation, but not sensitive to more general indicators of mental health risks. Specific brain-behavior links is the potential added value of the novel precision phenotyping task as compared to conventional parent-ratings. Subjective symptom screeners are known to have various limitations (e.g., limited test-retest reliability, moderate consistency between several responders, and high subjective bias [128]) risking the reliability of the observations. While the correlational analysis was more sensitive in capturing the neural mechanisms of typical attention functioning, among the paradigms tested, EPELI showed the greatest potential to evoke FC patterns that distinguish ADHD from TD. Together, these findings highlight EPELI’s potential for objective phenotyping, that could help in improving the reliability of the diagnostics [128] and in research support the initiatives for bridging the gap between phenotypes and biotypes in psychiatry [129–131].

## Limitations

Although the main results were largely consistent with our preregistered hypotheses, reproduction of the results in a bigger sample would be needed. The modest sample size might not provide enough statistical power to detect smaller effects that potentially could be present, for instance, in Resting State condition. Limited sample size might particularly affect comparisons between the conditions and analysis of task-general effects, where the participants are required to have intact data in all three conditions to be included in the analysis. That is, for this analysis, motion level needs to pass the criteria in all three conditions, which further reduced the number of participants that could be included. Even though our participant motion was at conventional and generally accepted level, the role of data quality and potential movement artefacts possibly contributing to the findings should be further examined in a larger study, as spurious motion artefacts could risk especially studies in children with hyperactive behaviors [132]. Together with limited sample size, high heterogeneity across ADHD participants, reflected in variability of the symptoms across the two core domains (inattention and impulsivity/hyperactivity), possible cultural and linguistic effects, previous medication use, and variability in the developmental trajectories could obscure association between FC and behavior in the ADHD group.

In the present study, we focused on NBS-framework and graph theory measures to keep the analyses coherent. However, a more elaborated analysis examining the paradigm-general effects is needed for exploring the possible effects that may not be captured by the present analyses. For example, the methods proposed by Cole and colleagues [84] allow to examine multitask FC and investigate specific architectural differences between intrinsic connectivity and task-specific connectivity. Implementing such or similar analysis would allow for a more comprehensive understanding of the FC patterns shared across different conditions. The selected brain parcellation, here the Brainnetome template [95], may also affect the result and therefore future studies could compare different brain parcellations to investigate the robustness of the findings. In addition, besides brain areas examined here there are other areas proposed to contribute to ADHD such as the brainstem [16,17], which should be acknowledged more carefully in future studies.

In administering the resting-state sequence last, there is a potential risk of carryover effects from task-related brain activity impacting the resting state connectivity measures. Although differences between pre- and post-task resting state FC have been demonstrated before in some specific conditions [133], the connectivity patterns of the pre- and post-task resting state scans are shown to be widely similar as both rely on intrinsic processes explaining majority of the signal changes [134]. Nonetheless, the fixed order of acquisition selected in the study may confound interpretations of resting-state FC, a considerably larger study with counter-balanced scan order would certainly be warranted.

Finally, further research is needed to explore how task demands affect FC patterns. Consideration of other VR tasks besides EPELI to cross-validate the relationship between ADHD symptoms and different naturalistic and non-naturalistic task types would be beneficial.

## Conclusions

This study demonstrates the opportunities of a novel VR-fMRI approach employing precision phenotyping and functional connectomics in studying brain functioning in developmental disorders. Using VR to emulate situations where the problems are manifested and simultaneously recording the ongoing brain activity, we revealed a widely distributed hyperconnectivity pattern involving especially subcortical areas that characterized children with ADHD. Moreover, we established an association between FC and objectively quantified clinical marker of ADHD in same participants that did not show brain-behavior associations between FC and conventional symptom screeners. Overall, our findings indicate importance of a deeper understanding of the coupling between dynamic brain states and behavioral states in ADHD and paying attention to ecological validity in future research. Such insight could help in building biologically-relevant psychiatric conceptualizations [129–131] and facilitate the development of precision tools for diagnostics.

## Supporting information

S1 File **S1 Appendix. Supplementary materials and methods.S1 Table. NBS EPELI FC group differences, edges.** The full list of edges included in the connected component which differed between the groups in EPELI task as indicated by NBS (Zalesky et al., 2010). The areas and lobes were defined based on Brainnetome Atlas (Jiang et al., 2013).**S2 Table. NBS EPELI FC group differences, nodes.** All nodes in the connected component, which was significantly different between the ADHD and TD group during EPELI. The nodes’ coordinates are given based on Brainnetome Atlas (Jiang et al., 2013).**S3 Table. NBS EPELI FC adjusted for task performance.** The nodes with the highest number of significant connections in the network detected by the NBS analysis of EPELI FC adjusted for task performance, in the group comparison ADHD>  TD group. Nodes with nodal degree >  5 are shown in the table.**S4 Table. Task Efficacy measure and EPELI FC in the TD group.** Nodal degree and location of the nodes in the network detected by the NBS analysis of correlation between EPELI Task Efficacy measure and EPELI FC in the TD group. All nodes are included in the table.**S5 Table. Task Efficacy measure and Video Viewing FC in the TD group.** Nodal degree and location of the nodes in the network detected by the NBS analysis of correlation between EPELI Task Efficacy measure and Video Viewing FC in the TD group. All nodes are included in the table.**S1 Fig. The EPELI FC network associated with Task Efficacy in EPELI game in the TD.** The NBS results were obtained with the primary statistic threshold of 3.5. Age and gender were included as covariates. The same analysis conducted with the threshold 4.0 is reported in the main text. There were no significant correlations observed with the threshold 4.5.**S2 Fig. The Video Viewing FC network associated with Task Efficacy in the EPELI game in the TD group.** The NBS results were obtained with the primary statistic threshold of 3.5. Age and gender were included as covariates. For the results with threshold 4.0 see S6 Fig in S1 File. There were no significant correlations observed with the threshold 4.5.**S3 Fig. Strongest FCs observed during the EPELI task**. First, the average FC matrix for each group and their difference were calculated. The resulted matrices were thresholded to represent 0.1%, 0.3% and 0.6% strongest connections respectively.**S4 Fig. The network showing stronger FC in the ADHD group than in the TD group during EPELI gameplay after correcting for general task performance effect**. No differences were observed in the opposite direction. The primary statistic threshold t =  3.5. (same as in other group comparison analyses reported in the paper). (A) anatomical representation, (B) alluvial diagram. L =  left; R =  right; a number indicates the count of significant links related to the area.**S5 Fig. The common nodes between the network indicated in NBS group comparison and the network indicated in correlational analysis of EPELI FC and Task Efficacy in the TD group.S6 Fig. The difference between FC in the ADHD and TD group in nodal degree during EPELI.S7 Fig. Strongest FCs observed during Video Viewing.** First, the average FC matrix for each group and their difference were calculated. The resulted matrices were thresholded to represent 0.1%, 0.3% and 0.6% strongest connections respectively.**S8 Fig. Strongest FCs observed during Resting State.** First, the average FC matrix for each group and their difference were calculated. The resulted matrices were thresholded to represent 0.1%, 0.3% and 0.6% strongest connections respectively.
(ZIP)
