# Supplementary material for: Real-world goal-directed behavior reveals aberrant functional brain connectivity in children with ADHD
Source: PLoS One. 2025 Mar 18;20(3):e0319746. doi: 10.1371/journal.pone.0319746 (PMC11918399; doi:10.1371/journal.pone.0319746)
Supplement: S1 File — S1 Appendix. Supplementary materials and methods.S1 Table. NBS EPELI FC group differences, edges. The full list of edges included in the connected component which differed between the groups in EPELI task as indicated by NBS (Zalesky et al., 2010). The areas and lobes were defined based on Brainnetome Atlas (Jiang et al., 2013).S2 Table. NBS EPELI FC group differences, nodes. All nodes in the connected component, which was significantly different between the ADHD and TD group during EPELI. The nodes’ coordinates are given based on Brainnetome Atlas (Jiang et al., 2013).S3 Table. NBS EPELI FC adjusted for task performance. The nodes with the highest number of significant connections in the network detected by the NBS analysis of EPELI FC adjusted for task performance, in the group comparison ADHD> TD group. Nodes with nodal degree > 5 are shown in the table.S4 Table. Task Efficacy measure and EPELI FC in the TD group. Nodal degree and location of the nodes in the network detected by the NBS analysis of correlation between EPELI Task Efficacy measure and EPELI FC in the TD group. All nodes are included in the table.S5 Table. Task Efficacy measure and Video Viewing FC in the TD group. Nodal degree and location of the nodes in the network detected by the NBS analysis of correlation between EPELI Task Efficacy measure and Video Viewing FC in the TD group. All nodes are included in the table.S1 Fig. The EPELI FC network associated with Task Efficacy in EPELI game in the TD. The NBS results were obtained with the primary statistic threshold of 3.5. Age and gender were included as covariates. The same analysis conducted with the threshold 4.0 is reported in the main text. There were no significant correlations observed with the threshold 4.5.S2 Fig. The Video Viewing FC network associated with Task Efficacy in the EPELI game in the TD group. The NBS results were obtained with the primary statistic threshold of 3.5. Age and gender were included as covariates. For the results [file pone.0319746.s001.zip › Supporting_information/S1_Appendix.docx]

**Supplementary Materials and Methods**

*Participants*

The volunteers were originally recruited from the participants of two previous behavioral studies (Seesjärvi et al., 2022; Merzon et al., 2022). This allowed us to invite participants that were not too hyperactive-impulsive during the gameplay (these symptoms are context-specific) and together with the parents estimate if the child could be able to perform the task in the scanner. At the same time the participants had already a good experience of the gameplay so that they knew what will happen during the scanning. An additional recruitment had to be conducted to reach the planned sample size according to the preregistration. To match the prior experience with the EPELI task with returning participants, new naïve participants first did the experimental session of the previous study that included EPELI (see, Merzon et al., 2022) and were then invited to the current study.

Participants were potentially eligible for the clinical group if they had ADHD diagnosis (F90) made by a licensed medical doctor. However, they were excluded if they had neurological or psychiatric disorders other than ADHD, except F93.89 (Emotional disorder with onset specific to childhood) and F93.9 (Unspecified childhood emotional disorder) which were allowed as comorbid diagnoses as they are commonly coexisting and closely linked to ADHD symptoms. One participant with G43 (migraine) was also accepted to the clinical group.

Data of some participants were excluded due to the following quality issues: two participants in the TD group and five participants in the ADHD group withdraw from the study during or before the first task due to discomfort; one participant in the TD group was excluded because of technical problems during the data acquisition; two participants in the ADHD group had medical conditions leading to exclusion (one had comorbid autistic spectrum disorder diagnosis and the other had myopia that could not be compensated with the available lenses); and one participant had excessive amount of movements in all the three tasks (see Data preprocessing section in the main text for more detail).

One participant in the ADHD group and one in the TD group stopped the experiment at the end of the gameplay, less than one minute to the actual end of the game. To ensure the data quality for these participants, 15 seconds were cut from the end of the respective scans, after that these participants were included in the analysis. Two more participants, both in the ADHD group, ended the experiment in the middle of the EPELI gameplay and were discarded from the analysis.

*Tasks*

*EPELI, additional information.* The main EPELI behavioral outcome measures defined in the original study of Seesjärvi and colleagues (2022) also included fifth parameter, Controller Motion, reflecting VR controller angular movement during task performance. This parameter was not included to the analysis of behavioral data due to incomparability between the trackball designed to minimize the movements in the scanner and a hand controller for VR with motion tracking that can be freely moved during the gameplay, which has been used in the previous ADHD studies (Seesjärvi et al., 2022; Merzon et al., 2022).

*Video Viewing, additional information.* Each gameplay video was followed by three questions (“How interested of this video were you?”, “How well could you focus on viewing the video?”, and “How difficult it was to follow the video?”) evaluated on a four-point Likert scale. Total duration of the Video Viewing task was approximately ten minutes. The order of the videos was pseudo-randomized so that two videos from a participant representing ADHD or TD group were never presented in a row. A ten second inter-trial washout period was included between the presentation of the videos to avoid carryover effects.

*Other tasks and questionnaires.* After scanning several questionnaires and additional tests were collected to examine any potential that could affect the results of the study. The children completed a shortened version of the Presence Questionnaire 3.0 (Witmer et al., 2005), a gaming experience questionnaire (Seesjärvi et al., 2022), a Continuous Performance Test (Rosvold et al., 1956), an instruction repetition test (Seesjärvi et al., 2022), which will be reported elsewhere, and two subtests (Vocabulary and Similarities) from fourth version of the Wechsler’s Intelligence Scale for Children were administered (WISC-IV; Wechsler, 2003). The severity of the ADHD symptoms was screened by the parents with ADHD Rating Scale-IV (ADHD-RS; DuPaul, 1998), and the Child Behavior Checklist (CBCL; Achenbach, 1991) was collected to screen other possible psychiatric symptoms.

*Data Preprocessing*

The mean number of volumes included in the analysis was 1141 (±152) for EPELI, 812 (±71) for Video Viewing, and 750 (±92) for Resting State. For EPELI, only the execution phase of each Task Scenario (excluding Practice Scenario) was included in the analysis.

For the Video Viewing task, the sequence was cut in separate videos and re-organized to the same order for all the participants. For the Video Viewing task, the acquired fMRI timeseries data was cut so that only the volumes during presentation of the videos were included and the data was re-organized to the same order for all the participants. These steps were performed at the end of the preprocessing pipeline before computing FC. This order for the preprocessing steps was chosen to ensure that there are no artifacts that could be introduced by preprocessing of a timeseries with signal “jumps” due to concatenation. This procedure could be considered as equivalent of calculating FC only during the segments corresponding to the video stimuli being on.

**References**

Achenbach T. M. (1991). Manual for the child behavior checklist/4-18 and 1991 profile. University of Vermont, Department of Psychiatry.

DuPaul G. J. (Ed.). (1998). ADHD rating scale-IV: Checklists, norms, and clinical interpretation. Guilford Press.

Merzon, L., Pettersson, K., Aronen, E. T., Huhdanpää, H., Seesjärvi, E., Henriksson, L., ... & Salmi, J. (2022). Eye movement behavior in a real-world virtual reality task reveals ADHD in children. *Scientific reports*, *12*(1), 20308.

Rosvold H. E., Mirsky A. F., Sarason I., Bransome E. D. Jr., Beck L. H. (1956). A continuous performance test of brain damage. *Journal of Consulting Psychology*, 20(5), 343–350. <https://doi.org/10.1037/h0043220>

Seesjärvi, E., Puhakka, J., Aronen, E. T., Lipsanen, J., Mannerkoski, M., Hering, A., ... & Salmi, J. (2022). Quantifying ADHD symptoms in open-ended everyday life contexts with a new virtual reality task. *Journal of Attention Disorders*, *26*(11), 1394-1411.

Wechsler, D. (2003). Wechsler intelligence scale for childrenFourth Edition (WISC-IV). *The Psychological Corporation.*

Witmer B. G., Jerome C. J., Singer M. J. (2005). The factor structure of the presence questionnaire. *Presence: Teleoperators and Virtual Environments*, 14(3), 298–312. <https://doi.org/10.1162/105474605323384654>
