# Supplementary material for: Real-world goal-directed behavior reveals aberrant functional brain connectivity in children with ADHD
Source: PLoS One. 2025 Mar 18;20(3):e0319746. doi: 10.1371/journal.pone.0319746 (PMC11918399; doi:10.1371/journal.pone.0319746)
Supplement: S1 File — S1 Appendix. Supplementary materials and methods.S1 Table. NBS EPELI FC group differences, edges. The full list of edges included in the connected component which differed between the groups in EPELI task as indicated by NBS (Zalesky et al., 2010). The areas and lobes were defined based on Brainnetome Atlas (Jiang et al., 2013).S2 Table. NBS EPELI FC group differences, nodes. All nodes in the connected component, which was significantly different between the ADHD and TD group during EPELI. The nodes’ coordinates are given based on Brainnetome Atlas (Jiang et al., 2013).S3 Table. NBS EPELI FC adjusted for task performance. The nodes with the highest number of significant connections in the network detected by the NBS analysis of EPELI FC adjusted for task performance, in the group comparison ADHD> TD group. Nodes with nodal degree > 5 are shown in the table.S4 Table. Task Efficacy measure and EPELI FC in the TD group. Nodal degree and location of the nodes in the network detected by the NBS analysis of correlation between EPELI Task Efficacy measure and EPELI FC in the TD group. All nodes are included in the table.S5 Table. Task Efficacy measure and Video Viewing FC in the TD group. Nodal degree and location of the nodes in the network detected by the NBS analysis of correlation between EPELI Task Efficacy measure and Video Viewing FC in the TD group. All nodes are included in the table.S1 Fig. The EPELI FC network associated with Task Efficacy in EPELI game in the TD. The NBS results were obtained with the primary statistic threshold of 3.5. Age and gender were included as covariates. The same analysis conducted with the threshold 4.0 is reported in the main text. There were no significant correlations observed with the threshold 4.5.S2 Fig. The Video Viewing FC network associated with Task Efficacy in the EPELI game in the TD group. The NBS results were obtained with the primary statistic threshold of 3.5. Age and gender were included as covariates. For the results [file pone.0319746.s001.zip › Supporting_information/S5_Table.docx]

**Table S5. Task Efficacy measure and Video Viewing FC in the TD group.**  Nodal degree and location of the nodes in the network detected by the NBS analysis of correlation between EPELI Task Efficacy measure and Video Viewing FC in the TD group. All nodes are included in the table.

| **Node Degree** | **Area^1^** | **Region^1^** | **Network^2^** |
| --- | --- | --- | --- |
| 45 | Orbital Gyrus R | Frontal Lobe | Sensory/somatomotor Mouth |
| 45 | Orbital Gyrus R | Frontal Lobe | Cingulo-opercular Task Control |
| 32 | Cingulate Gyrus R | Limbic Lobe | Fronto-parietal Task Control |
| 24 | Basal Ganglia R | Subcortical Nuclei | Subcortical |
| 24 | Basal Ganglia R | Subcortical Nuclei | Salience |
| 19 | Inferior Frontal Gyrus R | Frontal Lobe | Sensory/somatomotor Hand |
| 19 | Middle Temporal Gyrus R | Temporal Lobe | Uncertain |
| 19 | Middle Temporal Gyrus R | Temporal Lobe | Default mode |
| 18 | Superior Frontal Gyrus   R | Frontal Lobe | Uncertain |
| 18 | Superior Frontal Gyrus   R | Frontal Lobe | Sensory/somatomotor Hand |
| 17 | Orbital Gyrus L | Frontal Lobe | Sensory/somatomotor Hand |
| 17 | Orbital Gyrus L | Frontal Lobe | Sensory/somatomotor Mouth |
| 17 | Orbital Gyrus L | Frontal Lobe | Cingulo-opercular Task Control |
| 12 | Thalamus L | Subcortical Nuclei | Ventral attention |
| 12 | Thalamus L | Subcortical Nuclei | Cerebellar |
| 12 | Thalamus L | Subcortical Nuclei | Subcortical |
| 12 | Middle Frontal Gyrus  R | Frontal Lobe | Sensory/somatomotor Hand |
| 11 | Basal Ganglia L | Subcortical Nuclei | Salience |
| 11 | Basal Ganglia L | Subcortical Nuclei | Subcortical |
| 11 | lateral Occipital Cortex L | Occipital Lobe | Salience |
| 11 | lateral Occipital Cortex L | Occipital Lobe | Fronto-parietal Task Control |
| 10 | MedioVentral Occipital Cortex R | Occipital Lobe | Fronto-parietal Task Control |
| 7 | Cingulate Gyrus L | Limbic Lobe | Fronto-parietal Task Control |
| 7 | Cingulate Gyrus L | Limbic Lobe | Uncertain |
| 7 | Fusiform Gyrus R | Temporal Lobe | Default mode |
| 7 | Precuneus R | Parietal Lobe | Visual |
| 6 | Thalamus R | Subcortical Nuclei | Cerebellar |
| 6 | Superior Temporal Gyrus R | Temporal Lobe | Default mode |
| 5 | Middle Temporal Gyrus L | Temporal Lobe | Default mode |
| 5 | Inferior Frontal Gyrus L | Frontal Lobe | Sensory/somatomotor Hand |
| 5 | Hippocampus L | Subcortical Nuclei | Salience |
| 4 | Parahippocampal Gyrus R | Temporal Lobe | Default mode |
| 4 | Hippocampus R | Subcortical Nuclei | Salience |
| 4 | Inferior Parietal Lobule R | Parietal Lobe | Visual |
| 4 | Inferior Parietal Lobule R | Parietal Lobe | Ventral attention |
| 4 | Inferior Temporal Gyrus R | Temporal Lobe | Default mode |
| 4 | Middle Frontal Gyrus  L | Frontal Lobe | Sensory/somatomotor Hand |
| 3 | posterior Superior Temporal Sulcus  R | Temporal Lobe | Default mode |
| 3 | Precentral Gyrus R | Frontal Lobe | Cingulo-opercular Task Control |
| 3 | Superior Parietal Lobule R | Parietal Lobe | Memory retrieval? |
| 3 | Superior Parietal Lobule R | Parietal Lobe | Default mode |
| 3 | Inferior Parietal Lobule L | Parietal Lobe | Visual |
| 3 | Inferior Parietal Lobule L | Parietal Lobe | Memory retrieval? |
| 3 | lateral Occipital Cortex R | Occipital Lobe | Salience |
| 3 | MedioVentral Occipital Cortex L | Occipital Lobe | Fronto-parietal Task Control |
| 3 | Superior Frontal Gyrus   L | Frontal Lobe | Uncertain |
| 3 | Superior Frontal Gyrus   L | Frontal Lobe | Sensory/somatomotor Hand |
| 2 | Paracentral Lobule R | Frontal Lobe | Auditory |
| 2 | Amygdala R | Subcortical Nuclei | Salience |
| 2 | Inferior Temporal Gyrus L | Temporal Lobe | Default mode |
| 1 | Amygdala L | Subcortical Nuclei | Salience |
| 1 | Fusiform Gyrus L | Temporal Lobe | Default mode |
| 1 | Precuneus L | Parietal Lobe | Visual |

L = left; R = Right.

1 - Based on Brainnetome Atlas (Jiang et al., 2013).

2 - The network is assigned as defined by Power and colleagues (2011) by closest centroid
