# Supplementary material for: Real-world goal-directed behavior reveals aberrant functional brain connectivity in children with ADHD
Source: PLoS One. 2025 Mar 18;20(3):e0319746. doi: 10.1371/journal.pone.0319746 (PMC11918399; doi:10.1371/journal.pone.0319746)
Supplement: S1 File — S1 Appendix. Supplementary materials and methods.S1 Table. NBS EPELI FC group differences, edges. The full list of edges included in the connected component which differed between the groups in EPELI task as indicated by NBS (Zalesky et al., 2010). The areas and lobes were defined based on Brainnetome Atlas (Jiang et al., 2013).S2 Table. NBS EPELI FC group differences, nodes. All nodes in the connected component, which was significantly different between the ADHD and TD group during EPELI. The nodes’ coordinates are given based on Brainnetome Atlas (Jiang et al., 2013).S3 Table. NBS EPELI FC adjusted for task performance. The nodes with the highest number of significant connections in the network detected by the NBS analysis of EPELI FC adjusted for task performance, in the group comparison ADHD> TD group. Nodes with nodal degree > 5 are shown in the table.S4 Table. Task Efficacy measure and EPELI FC in the TD group. Nodal degree and location of the nodes in the network detected by the NBS analysis of correlation between EPELI Task Efficacy measure and EPELI FC in the TD group. All nodes are included in the table.S5 Table. Task Efficacy measure and Video Viewing FC in the TD group. Nodal degree and location of the nodes in the network detected by the NBS analysis of correlation between EPELI Task Efficacy measure and Video Viewing FC in the TD group. All nodes are included in the table.S1 Fig. The EPELI FC network associated with Task Efficacy in EPELI game in the TD. The NBS results were obtained with the primary statistic threshold of 3.5. Age and gender were included as covariates. The same analysis conducted with the threshold 4.0 is reported in the main text. There were no significant correlations observed with the threshold 4.5.S2 Fig. The Video Viewing FC network associated with Task Efficacy in the EPELI game in the TD group. The NBS results were obtained with the primary statistic threshold of 3.5. Age and gender were included as covariates. For the results [file pone.0319746.s001.zip › Supporting_information/S2_Table.docx]

**Table S2. NBS EPELI FC group differences, nodes.** All nodes in the connected component, which was significantly different between the ADHD and TD group during EPELI. The nodes’ coordinates are given based on Brainnetome Atlas (Jiang et al., 2013).

| **Node coordinates** | | | **Nodal Degree** |
| --- | --- | --- | --- |
| **x** | **y** | **z** |  |
| -27.0 | -4.0 | -20.0 | 12 |
| 11.0 | -85.0 | -9.0 | 12 |
| -7.0 | -80.0 | 9.0 | 11 |
| -52.0 | -50.0 | 11.0 | 6 |
| -15.0 | -71.0 | 51.0 | 6 |
| -17.0 | -25.0 | 6.0 | 6 |
| -11.0 | -21.0 | 14.0 | 4 |
| 9.0 | -14.0 | 14.0 | 4 |
| -6.0 | 7.0 | 38.0 | 4 |
| -38.0 | -61.0 | 46.0 | 4 |
| -49.0 | 36.0 | -3.0 | 4 |
| -23.0 | -48.0 | 65.0 | 4 |
| -6.0 | -11.0 | 5.0 | 3 |
| -13.0 | -20.0 | 74.0 | 3 |
| -23.0 | -12.0 | -20.0 | 3 |
| 51.0 | 37.0 | -1.0 | 3 |
| -32.0 | 14.0 | -35.0 | 3 |
| -33.0 | 23.0 | 45.0 | 3 |
| 28.0 | -4.0 | -20.0 | 3 |
| 31.0 | 15.0 | -33.0 | 3 |
| 16.0 | -64.0 | 25.0 | 2 |
| 57.0 | -44.0 | 39.0 | 2 |
| -53.0 | -31.0 | 22.0 | 2 |
| -6.0 | -63.0 | 51.0 | 2 |
| 18.0 | -9.0 | -30.0 | 2 |
| 35.0 | -42.0 | 54.0 | 2 |
| -8.0 | -24.0 | 41.0 | 2 |
| 16.0 | -85.0 | 35.0 | 2 |
| 22.0 | 1.0 | -36.0 | 2 |
| -51.0 | -33.0 | 41.0 | 2 |
| -16.0 | -31.0 | 2.0 | 2 |
| 48.0 | 35.0 | 13.0 | 2 |
| -18.0 | -1.0 | 65.0 | 2 |
| -4.0 | -10.0 | 8.0 | 2 |
| 6.0 | -21.0 | 41.0 | 2 |
| -54.0 | -32.0 | 13.0 | 2 |
| 30.0 | 37.0 | 36.0 | 2 |
| -49.0 | 5.0 | 30.0 | 2 |
| -32.0 | 4.0 | 55.0 | 2 |
| -46.0 | 13.0 | 23.0 | 2 |
| 54.0 | 24.0 | 12.0 | 2 |
| -52.0 | 23.0 | 11.0 | 2 |
| -27.0 | -30.0 | -10.0 | 1 |
| -23.0 | 37.0 | -17.0 | 1 |
| -6.0 | 34.0 | 21.0 | 1 |
| -7.0 | -94.0 | 0.0 | 1 |
| -13.0 | -68.0 | 12.0 | 1 |
| 19.0 | -59.0 | -7.0 | 1 |
| 7.0 | -76.0 | 11.0 | 1 |
| -23.0 | 7.0 | -4.0 | 1 |
| 56.0 | -40.0 | 13.0 | 1 |
| 54.0 | 14.0 | 12.0 | 1 |
| -12.0 | -82.0 | -11.0 | 1 |
| -5.0 | -41.0 | 31.0 | 1 |
| -18.0 | 23.0 | 53.0 | 1 |
| -31.0 | -89.0 | 11.0 | 1 |
| 32.0 | -84.0 | -11.0 | 1 |
| 20.0 | 5.0 | 64.0 | 1 |
| 33.0 | 8.0 | 54.0 | 1 |
| 42.0 | 22.0 | 3.0 | 1 |
| -49.0 | -7.0 | 39.0 | 1 |
| -32.0 | -9.0 | 58.0 | 1 |
| 33.0 | -7.0 | 57.0 | 1 |
| 54.0 | -24.0 | 10.0 | 1 |
| 51.0 | -3.0 | -1.0 | 1 |
| 64.0 | -29.0 | -13.0 | 1 |
| 58.0 | -15.0 | -10.0 | 1 |
| -46.0 | -27.0 | -26.0 | 1 |
| 31.0 | -61.0 | -14.0 | 1 |
| 43.0 | -49.0 | -19.0 | 1 |
| -16.0 | -60.0 | 62.0 | 1 |
| -33.0 | -47.0 | 50.0 | 1 |
| 22.0 | 26.0 | 51.0 | 1 |
| 55.0 | -10.0 | 15.0 | 1 |
| -38.0 | 5.0 | 4.0 | 1 |
| 4.0 | 6.0 | 38.0 | 1 |
| 22.0 | 9.0 | -1.0 | 1 |
